# Supplementary figures and images for: Association between periodontal disease due to Campylobacter rectus and cerebral microbleeds in acute stroke patients
Source: PLoS One. 2020 Oct 8;15(10):e0239773. doi: 10.1371/journal.pone.0239773 (PMC7544022; doi:10.1371/journal.pone.0239773)

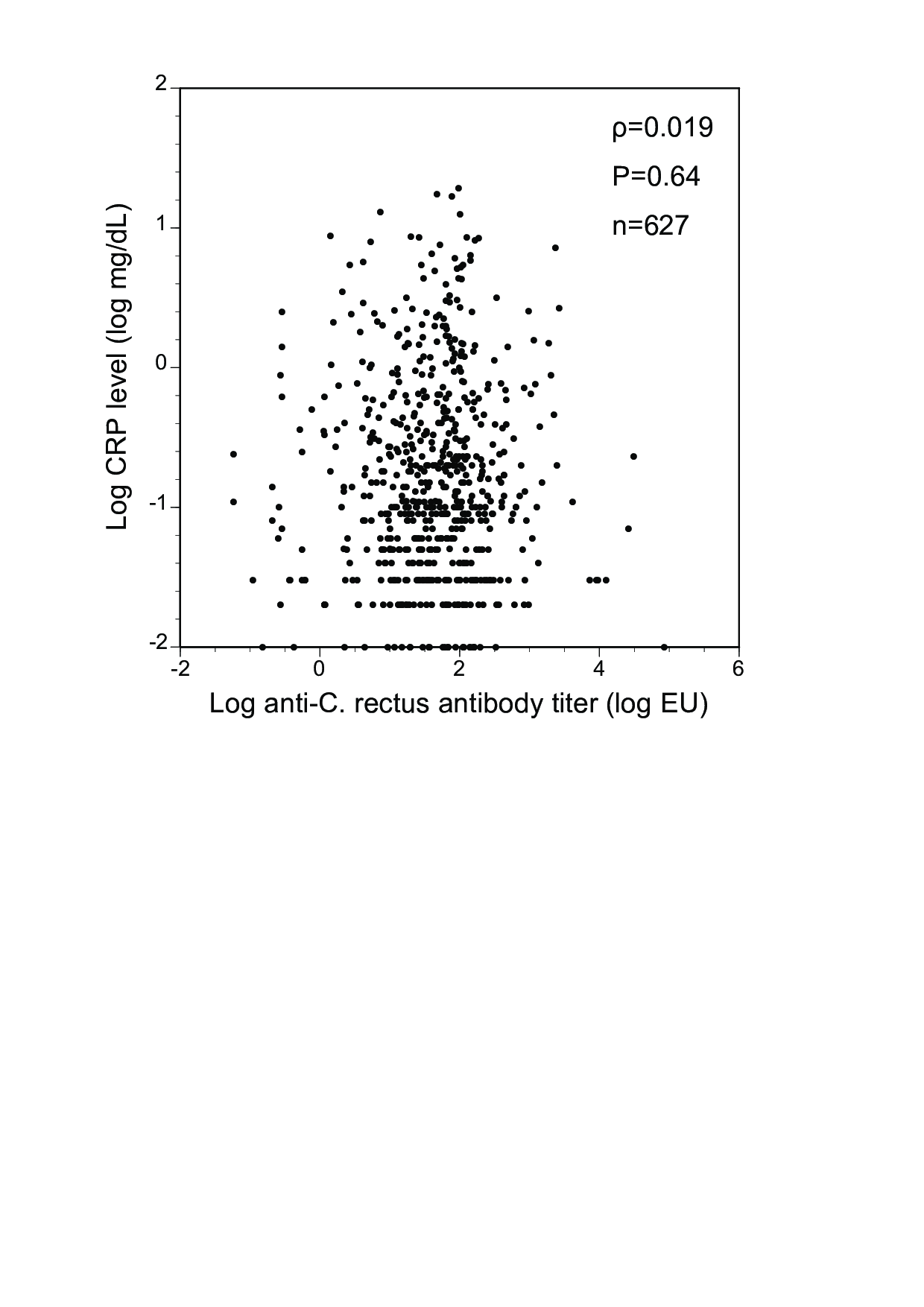

Supplement: S1 Fig — The analyses were performed using Spearman’s rank correlation coefficient. (TIF) [file pone.0239773.s001.tif]

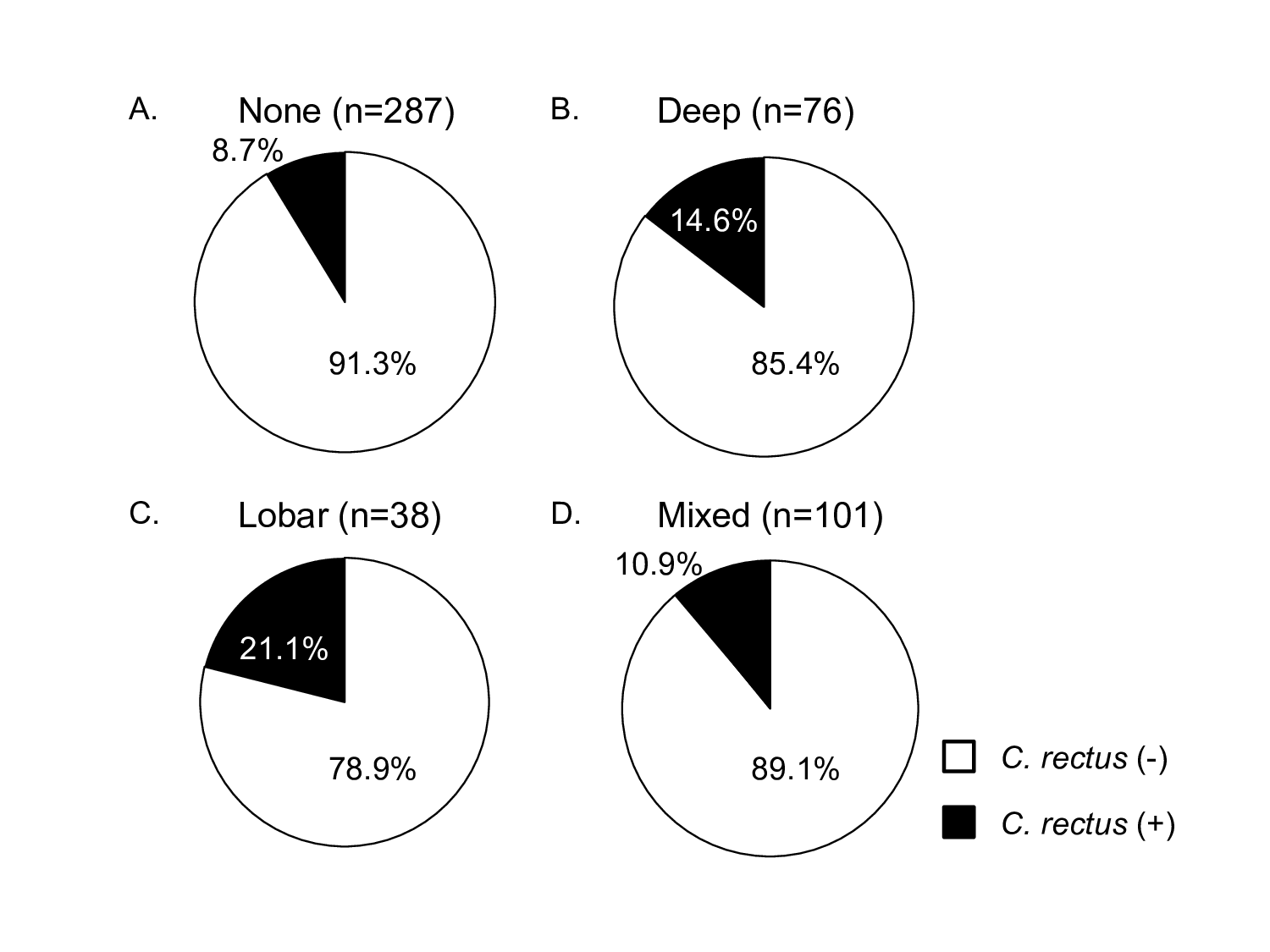

Supplement: S2 Fig — (TIF) [file pone.0239773.s002.tif]

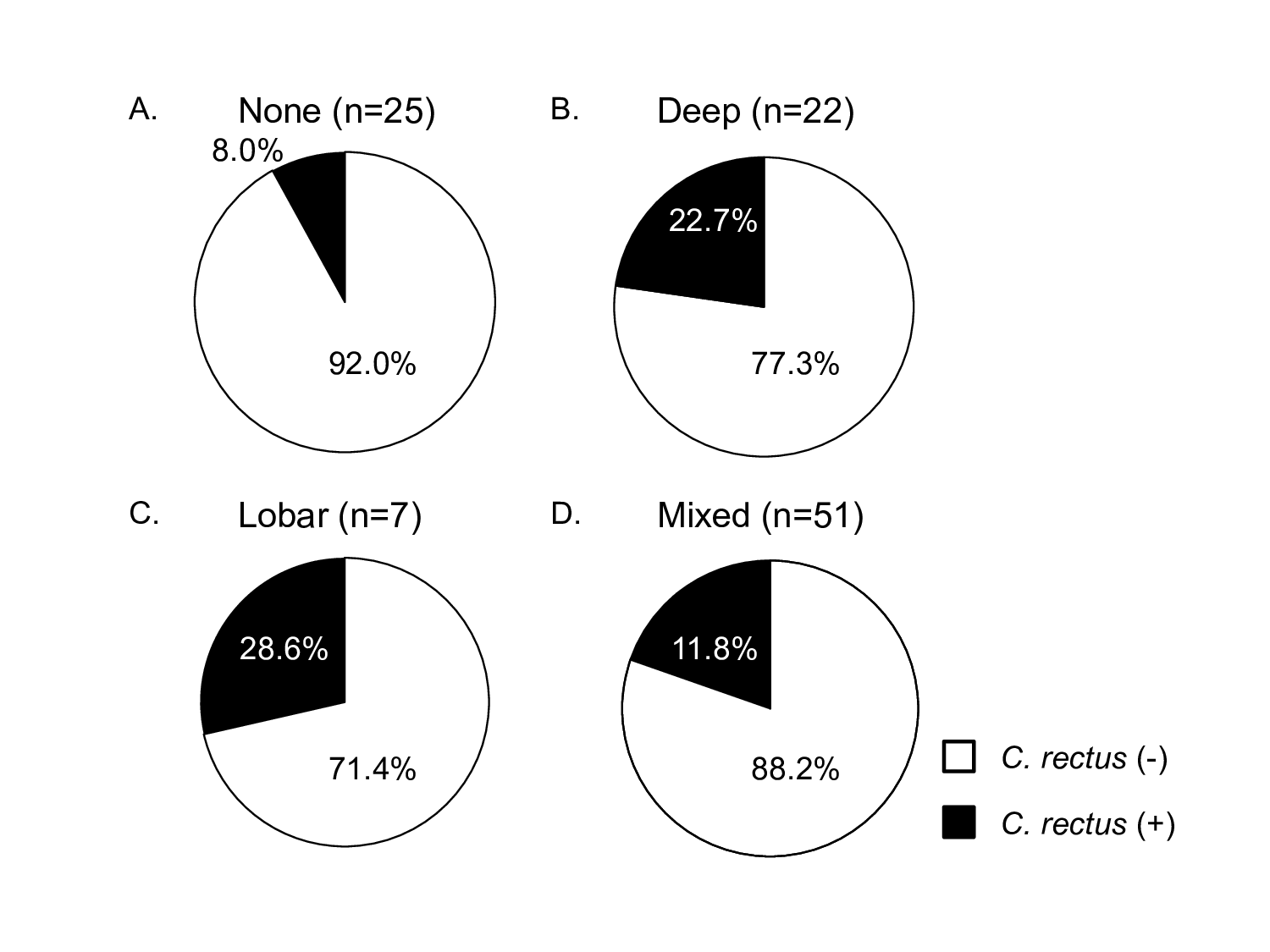

Supplement: S3 Fig — (TIF) [file pone.0239773.s003.tif]

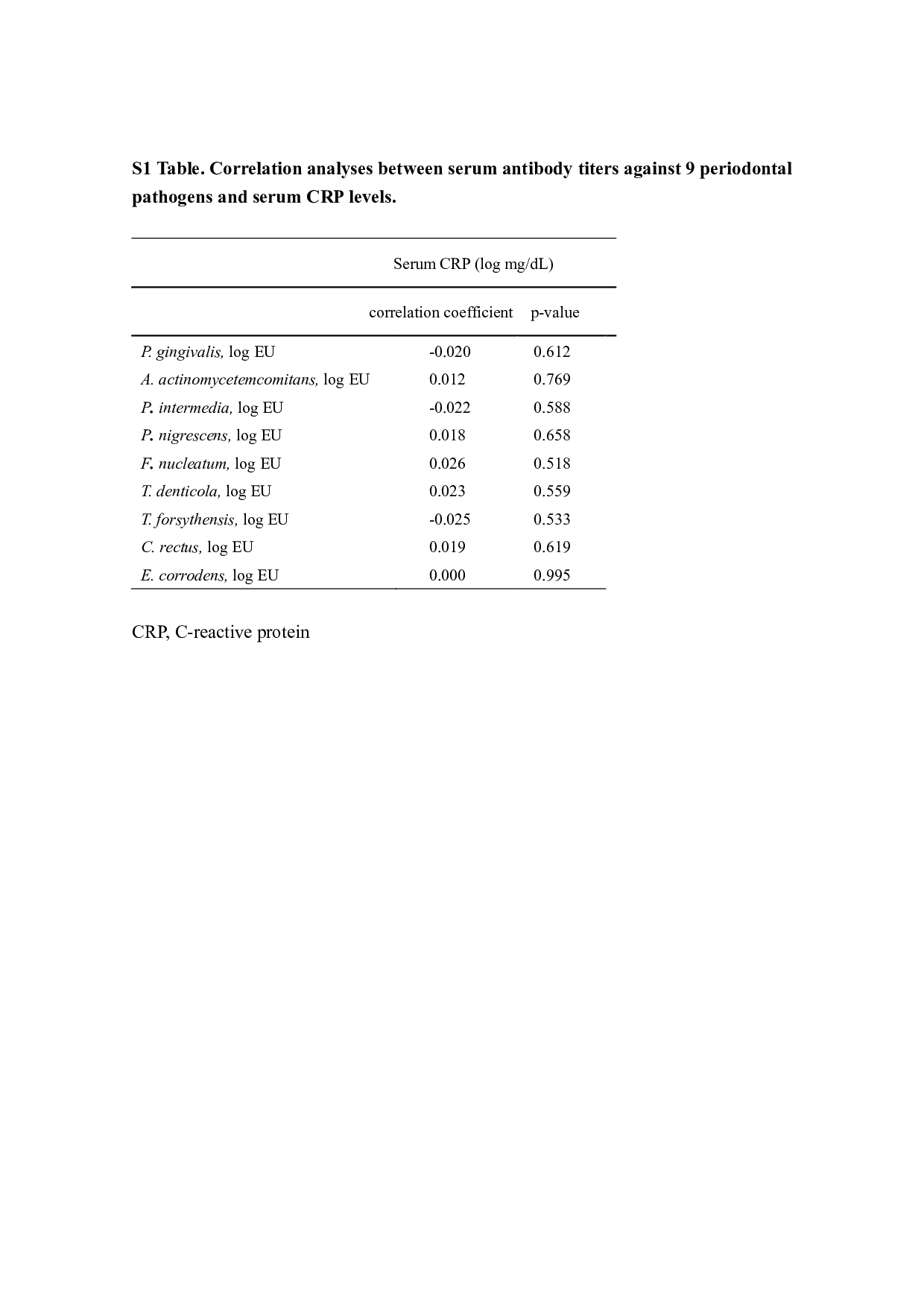

Supplement: S1 Table — The analyses were performed using Spearman’s rank correlation coefficient. (TIF) [file pone.0239773.s004.tif]

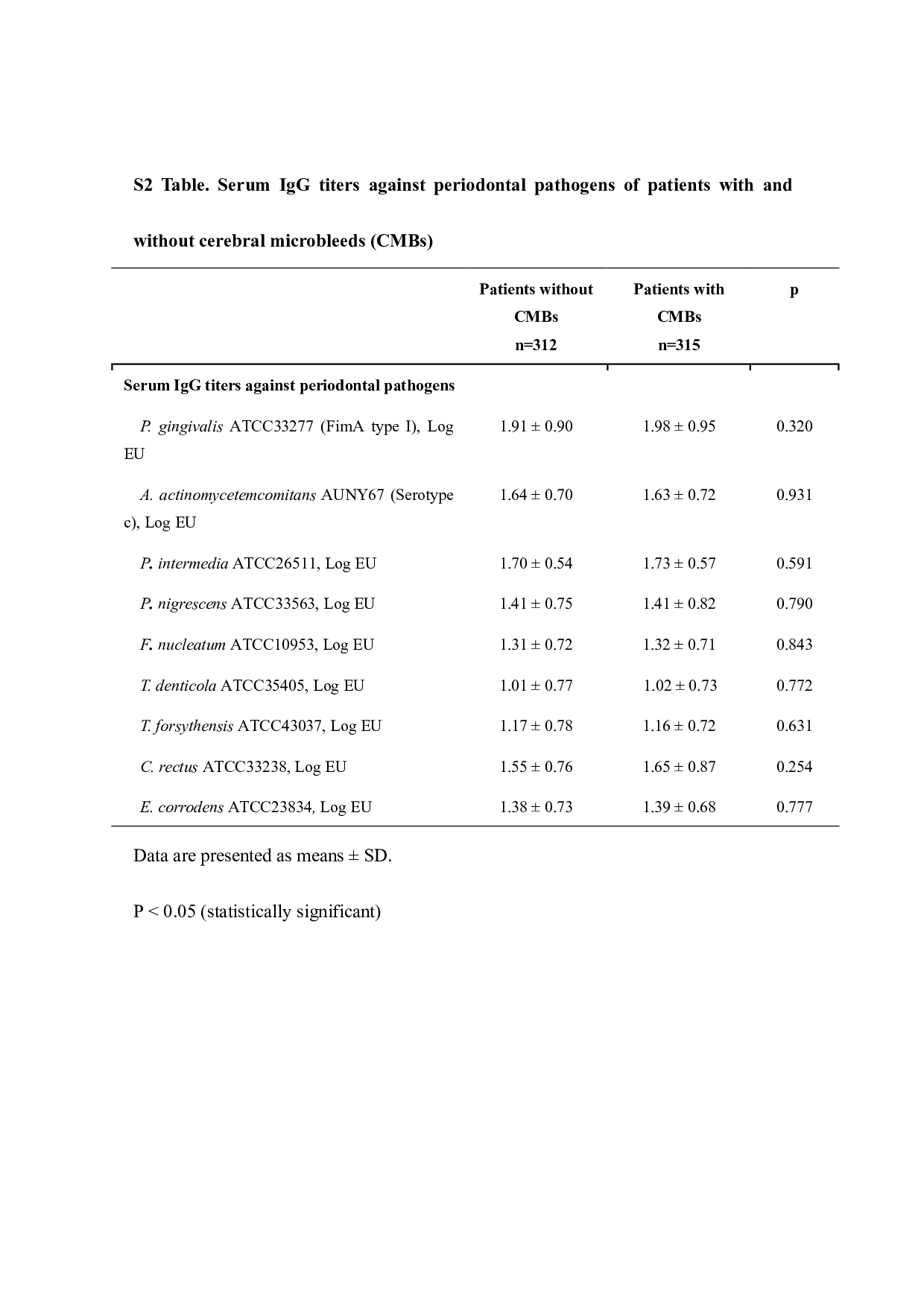

Supplement: S2 Table — (TIF) [file pone.0239773.s005.tif]
