# Supplementary material for: Association between periodontal disease due to Campylobacter rectus and cerebral microbleeds in acute stroke patients
Source: PLoS One. 2020 Oct 8;15(10):e0239773. doi: 10.1371/journal.pone.0239773 (PMC7544022; doi:10.1371/journal.pone.0239773)
Supplement: Attachment — Submitted filename: Response to Reviewers.docx [file pone.0239773.s007.docx]

Thank you again for reviewing our manuscript. We appreciate the insightful comments and advice of the reviewers. We have provided point-by-point responses to each of the comments below and highlighted the corresponding revisions in the manuscript document in red.

Response to the editor

Thank you for your comments. We have tried to respond to your comments as much as possible. We have highlighted the points of correction using red color letters in the revised text.

*We hope that our response meets your requests.*

1. *Please ensure that your manuscript meets PLOS ONE's style requirements, including those for file naming. The PLOS ONE style templates can be found at https://journals.plos.org/plosone/s/file?id=wjVg/PLOSOne_formatting_sample_main_body.pdf and* [*https://journals.plos.org/plosone/s/file?id=ba62/PLOSOne_formatting_sample_title_authors_affiliations.pdf*](https://journals.plos.org/plosone/s/file?id=ba62/PLOSOne_formatting_sample_title_authors_affiliations.pdf)

Response 1: Thank you for your suggestion. We will ensure and meet the PLOS ONE's style requirements. In addition, this paper was edited again by American Journal Experts. We add the Acknowledgments.(lines 348-351)

“**Acknowledgments**

This study was supported by research grants from the Japan Society for the Promotion of Science KAKENHI (Grant Numbers 17K17350, 17K17907, 18K10746, and 20K16579).“

2*. Thank you for including your competing interests statement;*

*"Dr. Maruyama reports grants and personal fees from Eisai, grants and personal fees from Pfiser, grants and personal fees from Takeda Pharmaceutical, grants and personal fees from Otsuka Pharmaceutical, grants and personal fees from Nihon Pharmaceutical, grants and personal fees from Teijin Pharma, grants from Shionogi, grants and personal fees from Fuji Film, grants and personal fees from Boehringer Ingelheim, grants and personal fees from Sumitomo Dainippon Pharma, grants and personal fees from Nihon Medi-Physics, grants and personal fees from Bayer, grants and personal fees from MSD, grants and personal fees from Daiichi Sankyo, grants and personal fees from Kyowa Kirin, grants and personal fees from Sanofi, grants and personal fees from Novartis, grants and personal fees from Kowa Pharmaceutical , grants and personal fees from Astellas Pharma, grants and personal fees from Japan Blood Products Organization, grants and personal fees from Mitsubishi Tanabe Pharma, personal fees from Ono pharmaceutical, personal fees from Biogen, personal fees from Bristol-Myers Squibb, grants from Mylan, outside the submitted work. All other authors declare that they have no conflicts of interest."*

*Please confirm that this does not alter your adherence to all PLOS ONE policies on sharing data and materials, by including the following statement: "This does not alter our adherence to PLOS ONE policies on sharing data and materials.” (as detailed online in our guide for authors* [*http://journals.plos.org/plosone/s/competing-interests*](about:blank)*).  If there are restrictions on sharing of data and/or materials, please state these. Please note that we cannot proceed with consideration of your article until this information has been declared.*

*Please include your updated Competing Interests statement in your cover letter; we will change the online submission form on your behalf.*

*Please know it is PLOS ONE policy for corresponding authors to declare, on behalf of all authors, all potential competing interests for the purposes of transparency. PLOS defines a competing interest as anything that interferes with, or could reasonably be perceived as interfering with, the full and objective presentation, peer review, editorial decision-making, or publication of research or non-research articles submitted to one of the journals. Competing interests can be financial or non-financial, professional, or personal. Competing interests can arise in relationship to an organization or another person. Please follow this link to our website for more details on competing interests:* [*http://journals.plos.org/plosone/s/competing-interests*](about:blank)

Response 2: Thank you for your suggestion. We added the following sentence in the Competing Interests section and included the Competing Interests statement in the cover letter. This does not alter our adherence to PLOS ONE policies on sharing data and materials.

Dr. Maruyama reports grants and personal fees from Eisai, grants and personal fees from Pfiser, grants and personal fees from Takeda Pharmaceutical, grants and personal fees from Otsuka Pharmaceutical, grants and personal fees from Nihon Pharmaceutical, grants and personal fees from Teijin Pharma, grants from Shionogi, grants and personal fees from Fuji Film, grants and personal fees from Boehringer Ingelheim, grants and personal fees from Sumitomo Dainippon Pharma, grants and personal fees from Nihon Medi-Physics, grants and personal fees from Bayer, grants and personal fees from MSD, grants and personal fees from Daiichi Sankyo, grants and personal fees from Kyowa Kirin, grants and personal fees from Sanofi, grants and personal fees from Novartis, grants and personal fees from Kowa Pharmaceutical , grants and personal fees from Astellas Pharma, grants and personal fees from Japan Blood Products Organization, grants and personal fees from Mitsubishi Tanabe Pharma, personal fees from Ono pharmaceutical, personal fees from Biogen, personal fees from Bristol-Myers Squibb, grants from Mylan which are unrelated to the submitted work. This does not alter our adherence to PLOS ONE policies on sharing data and materials. All other authors declare that they have no conflicts of interest.

3. *We note that you have included the phrase “data not shown” in your manuscript. Unfortunately, this does not meet our data sharing requirements. PLOS does not permit references to inaccessible data. We require that authors provide all relevant data within the paper, Supporting Information files, or in an acceptable, public repository. Please add a citation to support this phrase or upload the data that corresponds with these findings to a stable repository (such as Figshare or Dryad) and provide and URLs, DOIs, or accession numbers that may be used to access these data. Or, if the data are not a core part of the research being presented in your study, we ask that you remove the phrase that refers to these data.*

Response 3: Thank you for your suggestion. We added the Supplemental table (S1 Table) and legend. (lines 489-491)

“**S1 Table. Correlation analyses between serum antibody titers against 9 periodontal pathogens and serum CRP levels.** The analyses were performed using Spearman’s rank correlation coefficient”

Reply to the Reviewer 1

Thank you for your comments. We have tried to respond to your comments as much as possible. We have highlighted the points of correction using red color letters in the revised text.

We hope that our response meets your requests.

Reviewer #1: *The submission by Shiga et al. describes immunodetection of C. rectus at elevated levels in patients with cerebral microbleeds in acute stroke patients. The strengths of the manuscript are in the organization and presentation of data in the manuscript, a study that directly examines associations using human clinical data, and identification of C. rectus as on oral organism associated with microbleed in acute stroke patients. Areas of weakness include the lack of oral examination at the time of patient evaluation/serum collection, the lack of oral subgingival biofilm samples to identify the presence/absence of C. rectus in these oral samples, and reliance on antibody levels (ELISA) data only to indicate an association. Despite the clear omissions noted, this reviewer is weakly enthusiastic about the manuscript. Specific comments are as follows:*

1- *As identified by the authors, the strongest associated factor in acute stroke patients was hypertension and this aligns with the disease as an anticipated result. C. rectus is a significant factor in this study, but other periodontal organisms such as P. gingivalis, and A. actinomycetemcomitans have been associated with stroke previously in a study that had substantially more patients than the current study. What would be the precise limitation of enrolling fewer patients and observing differences in reported outcomes between the present study and the Pussinen et al. study that is referenced?*

Response 1:

Thank you for your comments. There are several major differences between the present study and the Pussinen et al. study. Pussinen et al. showed that seropositive of *P. gingivalis* were associated with the incident of stroke among general population (case-control study). In our study, we evaluated the association between various serum IgG titers of periodontal pathogens and the severity of cerebral small vessel disease among patients with acute stroke. Although our subjects were fewer than those in Pussinen et al. study, our findings can provide the novel insights between periodontal pathogens and the presence of cerebral small vessel based on the detail clinical and radiological findings for acute stroke patients.

2- *In the Methods, the number of patients should be identified, also, there is no mention of the numbers of total patients approached for exclusion in this study, or if there are any exclusion criteria.*

Response 2:

Thank you for your comments. In our study, consecutive acute stroke patients who were categorized as ischemic or hemorrhagic were registered. Patients who were not performed by MRI were excluded in this study.

We have revised the following sentences in the lines (96-98, 107-109).

“From January 2013 to April 2016, consecutive acute stroke patients who were categorized as ischemic or hemorrhagic were registered at Hiroshima University Hospital and Suiseikai Kajikawa Hospital.”

“Imaging analysis was performed with computed tomography or magnetic resonance imaging (MRI) in all patients for the diagnosis of ischemic stroke or intracerebral hemorrhage. In the present study, patients who did not undergo MRI were excluded because the detail radiological findings for cerebral small vessel disease were evaluated by MRI.”

In addition, we have revised the following sentences in the lines (179-181).

“Among the 690 consecutive acute stroke patients, 51 who did not undergo MRI because of contraindications or unsteadiness were excluded. Thus, a total of 639 patients (73.1 ± 12.9 years old, 285 males) were enrolled in this study.”

3- *There is essentially no description of the ELISA assay, determination of what type of antibodies (all, IgM, IgG, etc.) are being screed for in the sera. Further, it is not clear why pooled serum from only 5 health individuals was used to control? A similar number of control subject sera should be included in the study data, and there should be no pooling of samples as it is known that many periodontally healthy individual can have relatively different levels of circulation serum antibodies to periodontal disease-associated bacteria. This leads to potential concerns about statistical analysis of these serum antibody data. There is no description of the specific strains of each bacteria used in the present study. This must be included as it is known that organisms such as P. gingivalis have a fairly high degree of heterogeneity.*

Response 3:

Thank you for your comments.

We evaluated the serum IgG titers to periodontal pathogens, so we revised the phrase serum titer to serum IgG titer.

In the present study, serum samples from 5 healthy subjects were used as control data (Nishi et al. Clinical and Experimental Immunology, 2020). To obtain the additional data for larger control subjects, we should revise the methods of study and need an additional approval of Ethical Committee. Therefore, we adopted these data as controls.

We have added the limitation as follows (in lines 331-333).

“Third, there may be a lot of variability in the healthy controls due to the small number included (5 healthy subjects). This might lead to potential concerns about the statistical analysis of the serum antibody data.”

As a reviewer pointed, *Porphyromonas gingivalis* has several specific strains.

We also examined these specific strains; *Porphyromonas gingivalis* (fimA type I-V); *Aggregatibacter actinomycetemcomitans* (serotype a,b and c) ; *Fusobacterium nucleatum* (ATCC25586 and ATCC10953).

We selected *Porphyromonas gingivalis* (fimA type I) and *Aggregatibacter actinomycetemcomitans* (serotype c) on behalf of other strains, because there was no significant difference between small vessel disease (WMLs and CMBs) and all strains in this study. In addition, *Fusobacterium nucleatum* (ATCC10953) was selected because it was related to the stroke poor outcome in our previous study (Nishi et al. Clinical and Experimental Immunology, 2020).

We show the tables for the reviewer and selected species have under line.

Table. Comparison of antibody titers to periodontal pathogen in absence and presence CMB groups

| **Periodontal pathogen** | **Positive for periodontal pathogen, n (%)** | | |
| --- | --- | --- | --- |
|  | CMB absence  (n=312) | CMB presence  (n=315) | p-value |
| ***P. gingivalis* ATCC33277 (FimA type I)** | 36(11.5) | 44(14.0) | 0.403 |
| ***P. gingivalis* HW24D1 (FimA type II)** | 34(10.9) | 43(13.7) | 0.331 |
| ***P. gingivalis* 6/26 (FimA type III)** | 43(13.8) | 47(14.9) | 0.773 |
| ***P. gingivalis* W83 (FimA type IV)** | 35(11.2) | 46(14.6) | 0.234 |
| ***P. gingivalis* HNA99 (FimA type V)** | 39(12.5) | 44(14.0) | 0.638 |
| ***A. actinomycetemcomitans* ATCC29523 (Sero type a)** | 42(13.5) | 43(13.7) | 1.000 |
| ***A. actinomycetemcomitan*s Y4 (Sero type b)** | 42(13.5) | 32(10.2) | 0.217 |
| ***A. actinomycetemcomitans* AUNY67 (Sero type c)** | 40(12.8) | 32(10.2) | 0.318 |
| ***P. intermedia* ATCC26511** | 35(11.2) | 44(14.0) | 0.336 |
| ***P. nigrescens* ATCC33563** | 30(9.62) | 37(11.8) | 0.439 |
| ***F. nucleatum* ATCC25586** | 20(6.41) | 14(4.44) | 0.295 |
| ***F. nucleatum* ATCC10953** | 42(13.5) | 45(14.3) | 0.818 |
| ***T. denticola* ATCC35405** | 49(15.7) | 52(16.5) | 0.828 |
| ***T. forsythensis* ATCC43037** | 43(13.8) | 45(14.3) | 0.909 |
| ***C. rectus* ATCC33238** | 27(8.65) | 46(14.6) | 0.025* |
| ***E. corrodens* ATCC23834** | 38(12.2) | 42(12.8) | 0.720 |

Table. Comparison of antibody titers to periodontal pathogen in the severity of WMLs

| **Periodontal pathogen** | **The severity of WMLs for periodontal pathogen, n (%)** | | |
| --- | --- | --- | --- |
|  | mild WMLs  (n=442) | severe WMLs  (n=197) | p-value |
| ***P. gingivalis* ATCC33277 (FimA type I)** | 56 (12.7) | 24 (12.2) | 0.898 |
| ***P. gingivalis* HW24D1 (FimA type II)** | 54 (12.2) | 23 (11.7) | 0.896 |
| ***P. gingivalis* 6/26 (FimA type III)** | 65 (14.7) | 26 (13.2) | 0.713 |
| ***P. gingivalis* W83 (FimA type IV)** | 53 (12.0) | 28 (14.2) | 0.442 |
| ***P. gingivalis* HNA99 (FimA type V)** | 59 (13.4) | 25 (12.7) | 0.899 |
| ***A. actinomycetemcomitans* ATCC29523 (Sero type a)** | 63 (14.3) | 24 (12.2) | 0.534 |
| ***A. actinomycetemcomitan*s Y4 (Sero type b)** | 56 (12.7) | 20 (10.2) | 0.428 |
| ***A. actinomycetemcomitans* AUNY67 (Sero type c)** | 55 (12.4) | 18 (9.1) | 0.281 |
| ***P. intermedia* ATCC26511** | 52 (11.8) | 27 (13.7) | 0.516 |
| ***P. nigrescens* ATCC33563** | 49 (11.1) | 19 (9.6) | 0.677 |
| ***F. nucleatum* ATCC25586** | 24 (5.4) | 10 (5.1) | 1.000 |
| ***F. nucleatum* ATCC10953** | 53 (12.0) | 34 (17.3) | 0.081 |
| ***T. denticola* ATCC35405** | 71 (16.1) | 30 (15.2) | 0.816 |
| ***T. forsythensis* ATCC43037** | 58 (13.1) | 30 (15.2) | 0.534 |
| ***C. rectus* ATCC33238** | 47 (10.6) | 26 (13.2) | 0.349 |
| ***E. corrodens* ATCC23834** | 58 (13.1) | 23 (11.7) | 0.700 |

4- *Page 14 (lines 209-211). These data are different than what is presented on page 10. Please clarify.*

Response 4:

Table 1 showed the all date evaluated the serum IgG titer to periodontal pathogens. 639 patients were evaluated for cerebral small vessel disease on MRI images. 639 patients were evaluated WMLs on FLAIR image, however, 12 patients could not get a clear T2* image because of artifacts caused by body movement. We could not evaluate the numbers and location of CMBs in these 12 patients.

Thus, we described that “Of the 639 evaluated patients, 12 were excluded because of artifacts caused by body movement during the evaluation of CMBs on T2*WI. Hence, 627 patients were analyzed (ischemic, n = 522 and hemorrhagic, n = 105) (lines 212-214).”

5- *Page 20 (lines 291-294). With the knowledge of S. mutans associations association with CMBs, it is curious that in the present manuscript that S. mutans levels of specific antibody were not assessed in the patients examined? Why only the focus on the subgingival group? Indeed, this reviewer thinks that having these data would make for an interesting and important comparison to prior work in this area.*

Response 5:

In general, *Cnm*-positive *S. mutans* DNA of each strain was extracted by oral samples (saliva or dental plaque). *S.mutans* and *cnm* gene encoding CBP was screened using polymerase chain reaction techniques. (Nomura et al. J Med Microbiol. 2009, Sato et al. J Dent Res. 2004) In our study, we could not evaluate the presence of *Cnm*-positive *S. mutans* based on saliva or dental plaque using these methods. In future, we would like to elucidate the association between S. mutans, periodontal pathogen, and CMBs using serum and oral samples.

6- *CRP was the only inflammatory marker examined. Numerous studies support that pro-inflammatory cytokines serve as important markers of overall systemic inflammation. Further, systemic inflammation is liked to IL-1 (Sobowale et al. Stroke 2016. 47:2160-67). Evaluation of the levels of other inflammatory markers would improve understanding of potential underlying mechanisms in this association, and are thus suggested.*

Response 6:

Thank you for your comments. We have revised the following sentences in the lines (321-327).

“First, this study targeted acute ischemic stroke patients, and various factors, such as systemic inflammation involving CRP or several cytokines, might therefore affect the association between serum antibody titers and both periodontal disease and CSVD (Sobowale et al. Stroke 2016. 47:2160-67, Nambi et al. Stroke 2009. 40(2):376-81.).[34, 35] Although we could not evaluate cytokine levels, we found no significant associations between serum antibody titers and CRP levels (S1 Table). Hence, the association between serum periodontal antibody titers and CSVD is likely not due to systemic inflammation alone.”

Reviewer #2: *The authors aim to evaluate the associations between periodontal pathogens with the presence or severity of cerebral microbleeds and white matter lesions in acute stroke patients. There are good numbers of observational studies that periodontal disease is highly associated with the incidence of cardioembolic and thrombotic stroke. Understanding the whether any specific types of pathogen related to periodontitis is associated with the incidence of stroke is highly crucial.
The experimental design is well organized, and the results might be impactful to the field of stroke and cerebrovascular disorders.*

 Reply to the Reviewer 2

Thank you for your comments. We have tried to respond to your comments as much as possible. We have highlighted the points of correction using red color letters in the revised text.

We hope that our response meets your requests.

*I have only one minor suggestion.
It is a well written and taught study, which would be impactful in the field. However, the quality/number of the figures are quite low, I will suggest improving numbers of figures including a range of descriptive T2*-weighted MRI for white matter lesion and cerebral microbleeds. It would necessary if authors have correlative illustrations between severity of lesions in MRI and of periodontal pathogens. This figure would be increase quality of presentation.*

Response 1:

Thank you for your comments. The image quality was changed according to PLOS ONE standards.

In the present study, the prevalence of positive serum antibody titers against *C. rectus* is independently associated with the presence of CMBs after adjusting for several confounding factors. However, the patients with positive serum antibody titers against *C. rectus* had slightly higher, but not significantly, numbers of CMBs than those without (median [interquartile range], 1 [0–4] vs. 0 [0–3], P = 0.077, Fig 1). Increased serum IgG titer against *C.rectus* were also not associated with increased numbers of cerebral microbleeds (CMBs) (Spearman correlation analysis, ρ = 0.038, P = 0.347). As noted in the discussion, we speculated that these results may indicate that a positive serum antibody titer against *C. rectus* is associated with the initial occurrence of CMBs but not with an increase in the number of CMBs.
