# Supplementary material for: Association between periodontal disease due to Campylobacter rectus and cerebral microbleeds in acute stroke patients
Source: PLoS One. 2020 Oct 8;15(10):e0239773. doi: 10.1371/journal.pone.0239773 (PMC7544022; doi:10.1371/journal.pone.0239773)
Supplement: Attachment — Submitted filename: Response to Reviewers.docx [file pone.0239773.s008.docx]

 Thank you again for reviewing our manuscript. We appreciate the insightful comments and advice of the reviewers. We have provided point-by-point responses to each of the comments below and highlighted the corresponding revisions in the manuscript document in red.

Response to the editor

Thank you for your comments. We have tried to respond to your comments as much as possible. We have highlighted the points of correction using red color letters in the revised text.

We hope that our response meets your requests.

*Reviewer #1: The revised submission PONE-D-20-11864R1 has made significant improvements from the interesting original submission. As few items are felt to require additional attention.

1- In the limitations of the study, the lack of a clinical periodontal exam of all patients included in this study should be listed as limitation #1 in the listing of limitations.*

Response 1: Thank you for your suggestion. We have added the limitation as follows (in lines 326-328).

First, we could not evaluate the clinical periodontal examination of all patients. It is not fully clear how serum antibody titers against periodontal disease are influenced by the presence or severity of periodontal disease.

2- In the limitations of the study, the lack of a similar number of controls, and the handling of the 5 samples (as pooled) should be moved to limitation #2 in the listing of limitations described in the discussion.

Response 2: Thank you for your comments. We have changed the limitation as follows (in lines 335-337).

Third, there may be a lot of variability in the healthy controls due to the small number included (5 healthy subjects). This might lead to potential concerns about the statistical analysis of the serum antibody data.

3- This reviewer thanks the authors for identifying that IgG levels were determined. However, the indication of “titer” is not presented in any of the tables, rather “% positive” was used to compare between clinical groups. This reviewer suggests that the serum titer +/- error for each bacterium for each of the patient/serum samples examined be included in each of the tables reporting titer information.

Response 3: Thank you for your comments.We added the data of serum IgG titer against each periodontal pathogen in S2 Table. There is no significant difference in the association with serum IgG titer against *C. rectus* and the presence of CMBs. Therefore, we added the limitation and supporting information as follows (in lines 337-342 and 501-502).

“Fourth, we did not focus on increased levels of titers of each periodontal pathogen, although we focused on the positive rate. In fact, there is no significant difference in the association with serum IgG titer against *C. rectus* and the presence of CMBs (S2 table). The limitation of this study was that we revealed only the association with positive rate of serum IgG titers and presence of CMBs.”

**“S2 Table.** Associations between the presence of CMBs and serum IgG titer against periodontal pathogens.”

Minor points:
1- Please define “WMLs” on first use in the text.

Thank you for your comments. We have added the sentence as follows (in lines 137-138).

In this study, we defined the WMLs as periventricular hyperintensity.

.

2- This reviewer thanks the authors for including the strain examination data for the various bacterial lysates in the response to reviewer comments. Please apply the strain information throughout the Methods, and presented data (all relevant Tables).

Thank you for your comments. We have changed the sentence as follows (in lines 154-159) and added the strains in Table 2 and Table 3.

*P. gingivalis* ATCC33277 (FimA type I), *A. actinomycetemcomitans* AUNY67 (Serotype c), *P. intermedia* ATCC26511, *Prevotella nigrescens (P. nigrescens)* ATCC33563, *F. nucleatum* ATCC10953, *Treponema denticola* *(T. denticola)* ATCC35405, *Tannerella forsythensis (T. forsythensis)* ATCC43037, *Campylobacter rectus (C. rectus)* ATCC33238, and *Eikenella corrodens (E. corrodens)* ATCC23834.

3- Table 2 and Table 3 titles. Please change “antibody” to “IgG”.

Thank you for your comments. We revised the table 2 and table 3 titles.

4- In all tables, please identify using symbols, those data that demonstrate significant differences between groups. Please identify and describe the symbol and test used in each of the corresponding figure legends, table descriptions, respectively.

Thank you for your suggestion. We added symbols to explain the data that demonstrate significant differences between groups.
